# Use of Sulphur

**Published:** 1883-07

**Authors:** 


### USE OF SULPHUR.

In answer to our correspondent from Austin, Texas, who desires us to *give the full effects of sulphur on sheep in wet weather*, we would say, use it not at all in wet weather. As upon the human patient, externally, so upon sheep or other domesticated animals it should be used only upon a quarter of the body at one time in the form of ointment. When it is deemed necessary to use sulphur in treating animals, they should be kept in warm, airy stables, or, if in the open air, warm, sunny exposures should be sought for them.

Those who use sulphur for the destruction of lice can succeed better with carbolic acid or white precipitate ointment of the strength of a drachm to the ounce of lard.

Sulphur has produced rheumatism in man and beast when long and injudiciously used.

